# Added-Value of Endometrial Biopsy in the Diagnostic and Therapeutic Strategy for Pelvic Actinomycosis

**DOI:** 10.3390/jcm9030821

**Published:** 2020-03-18

**Authors:** Julie Carrara, Blandine Hervy, Yohann Dabi, Claire Illac, Bassam Haddad, Dounia Skalli, Gregoire Miailhe, Fabien Vidal, Cyril Touboul, Charlotte Vaysse

**Affiliations:** 1 Service de Gynécologie Obstétrique, Université Paris Est, Paris XII, Hôpital Intercommunal de Créteil, 94000 Créteil, France; julie.carrara@gmail.com (J.C.); yohann.dabi@gmail.com (Y.D.); bassam.haddad@chicreteil.fr (B.H.); Dounia.Skalli@chicreteil.fr (D.S.); Gregoire.Miailhe@chicreteil.fr (G.M.); cyril.touboul@gmail.com (C.T.); 2 Service de Chirurgie générale et gynécologique, Université de Toulouse, CHU de Toulouse, UPS, 31059 Toulouse, France; blandine.hervy@gmail.com (B.H.); vidal.fabien@chu-toulouse.fr (F.V.); 3 Service d’Anatomie-Pathologie, Université de Toulouse, CHU de Toulouse, 31059 Toulouse, France; illac.claire@iuct-oncopole.fr

**Keywords:** pelvic actinomycosis, endometrial biopsy, pelvic abscess, intra-uterine device, antibiotics

## Abstract

The particularity of pelvic actinomycosis lies in the difficulty of establishing the diagnosis prior to treatment. The objective of this retrospective bicentric study was to evaluate the pertinence and efficacy of the different diagnostic tools used pre- and post-treatment in a cohort of patients with pelvic actinomycosis. The following data were collected: clinical, paraclinical, type of treatment, and the outcome and pertinence of the two diagnostic methods, bacteriological or histopathological, were evaluated. Twenty-seven women were included, with a pre-treatment diagnosis proposed for 66.7% (*n* = 18) of them. The diagnosis was established in 13.6% (*n* = 3) of cases through bacteriological samples, and in 93.8% (*n* = 15) of cases through histopathological samples, with endometrial biopsy positive in 100% of cases. The treatment was surgical with antibiotics for 55.6% (*n* = 15) of patients, medical with antibiotic therapy for 40.7% (*n* = 11) of patients, and surgical without antibiotics for one patient. All patients achieved recovery without recurrence, with a median follow-up of 96 days (4–4339 days). Our study suggested an excellent performance of histopathological analysis, and in particular endometrial biopsy, in the diagnosis of pelvic actinomycosis. This tool allowed early diagnosis and, in some cases, the use of antibiotic therapy alone, making it possible to avoid surgery.

## 1. Introduction

Actinomycosis is a chronic bacterial infection of which the causative agent belongs to the large family of Actinomyces [1,2]. This saprophytic bacterium can be found on the human body in the gastrointestinal, oropharyngeal, and urogenital tracts [1]. Generally non-pathogenic, it can be responsible for infection by contiguity in case of erosion of the mucous membranes, evolving chronically and insidiously because of its weak virulence [3]. In the pelvis, colonization by Actinomyces is frequent, does not necessarily lead to infection, and is favored by the use of an intra-uterine device (IUD; 1% to 2% positive swabs after 1 year of use, and 5–10% positive after 5 years) [4,5,6] (1.6% to 44%) [7], the alkalization of vaginal secretions, and immunodepression [8]. Its presence at a cervical (Pap) smear is not synonymous with infection, and is not predictive of the later development of pelvic inflammatory disease (PID) [8]. Only the use of a copper IUD and the duration of its use (7 years on average) [9,10], gynecological and digestive surgeries [11], and a state of immunodepression [12] have been incriminated in the process of pathological development of the bacterium. 

The particularity of this disease lays in the difficulty of establishing its diagnosis prior to treatment. Its appearance in imaging is non-specific and heterogeneous, often with a finding of a tissue mass that may be abscessed, developed to the detriment of the uterus and/or the annexes, and extending to adjacent organs. Compressions (hydroureteronephrosis) or infiltrations of the fascia and the viscera are sometimes described [13,14]. In over half of cases, bacteriological samples are negative, and thus do not point to an infectious cause [2]. In this context, the hypothesis of an advanced ovarian neoplasm is most often adopted without a histopathological analysis being done, as one is not recommended [15,16]. Extensive surgery of the oncological type is often performed, and the diagnosis is established a posteriori with reference to the definitive pathological results. Thanks to biopsies, some cases have been diagnosed before any treatment, making it possible to avoid surgery and to attempt medical treatment by prolonged antibiotic therapy, which has been proven to be effective [17]. Only one study in the literature showed a benefit of cytology in pre-operative diagnosis [18], and none have evaluated the performance of different diagnostic methods. 

The objective of our study was to evaluate the pertinence and efficacy of the different diagnostic tools used pre- and post-treatment in a cohort of patients with pelvic actinomycosis (PA). The medical and/or surgical efficacy of treatments is also described.

## 2. Materials and Methods

We conducted a retrospective, observational, bicentric study that included all cases of PA treated at the University Hospital Center of Toulouse and at the Intercommunal Center of Créteil between January 2000 and December 2018. The study protocol was approved by the Institutional Review Board, the Comité d’éthique de la recherche en obstétrique et gynécologie (CEROG) (N° 2020-GYN-0204, date of approval March 5th, 2020). The diagnosis of PA was based on the association of one of several clinical criteria (fever, metrorrhagia, leukorrhea, pelvic pain) and paraclinical criteria (inflammatory syndrome, imaging abnormalities) with bacteriological and/or histopathological confirmation. All data were collected retrospectively via computerized and archived records. 

All patients with a finding of Actinomyces in the abdominal–pelvic region, determined via histopathological and/or bacteriological examination and meeting the above definition, were included. All patients carrying an asymptomatic Actinomyces-like organism (ALO) were excluded, as were those with a possible differential diagnosis: proven neoplasia or proven sexually transmitted disease. Phone contact was made with patients in the case of missing data concerning follow-up and the evolution of symptoms, and to identify recurrences or complications. 

The following clinical data were collected: age, obstetric–gynecological and digestive history, immunodepression status (immunosuppressive treatment, diabetes), the presence of an IUD and duration of use, fever, metrorrhagia, pelvic pain, and leukorrhea. Concerning biologic paraclinical data, the following were noted: the presence of an inflammatory syndrome (hyperleucocytosis associated with an increase in C-reactive protein (CRP)), the assay of the CA125 marker, positive results of diagnostic tests (bacteriological samples: vaginal smear, endocervix, hemocultures, IUD per-operative; cytological samples: targeted biopsy, endometrial biopsy, Pap smear, operative specimen). With regard to imaging examinations, conclusions of ultrasound, computed tomography scanners (CT scans), and MRIs were collected. Data on treatment choices (surgeries/antibiotics), their duration, and means of administration, as well as events (recoveries, relapses, sequelae) were collected. 

Due to the great heterogeneity of the clinical pictures, the patients were classified into six categories according to the type of infiltration they presented at imaging: endometritis, tubo-ovarian abscess, infiltrating mass without involvement of adjacent organs, infiltrating mass with involvement to adjacent organs, chorioamnionitis, or unknown. Lacking recommendations, the choice of diagnostic tool was left up to the clinician according to the clinical picture. These tools were grouped into two categories: bacteriological (vaginal and endocervical swab, bacteriological culture of the IUD, and blood cultures) and histopathological (Pap smear, endometrial biopsy, and biopsy of the lesion under imaging guidance). Among the bacteriological tests, hemocultures were performed in cases of fever of > 38.5 °C (aerobic and anaerobic). The vaginal, endometrial, and surgical samples were obtained with standard swabs. The IUD was removed at the first visit or at surgery and sent for bacteriological analysis in a standard aerobic milieu, as were the per-operative samples. Among the histopathological tests, cervical smears were taken using a cytobrush and preserved in a liquid milieu. Endometrial biopsies were performed using a Pipelle de Cornier and samples were preserved in liquid milieu during consultation.

**Figure 1:**
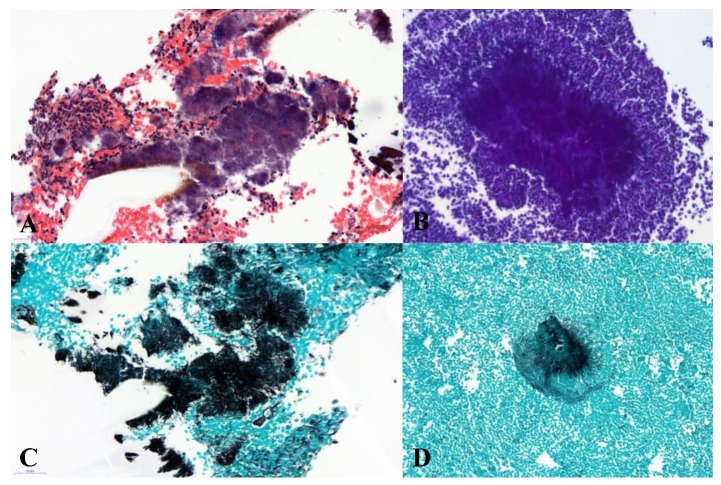
Different stains allowed the diagnosis of actinomycosis in histopathology. (**A**) Cluster of actinomyces with grains of sulfur in standard stain (hematoxylin and eosin, ×400); (**B**) periodic acid Schiff stain (×200); (**C**) radial filaments, Grocott stain (×400); and (**D**) cluster of Actinomyces, Grocott stain (×200).

The latter device is a disposable, sterile, (consists of a transparent, flexible, perforated sheath) single-use aspiration curette intended to be used to obtain a histological biopsy of the lining of the uterus or to take a uterine menstrual sample for microscopic analysis or culture. No anesthesia was required. In addition to the standard staining with hematoxylin and eosin, a stain with a periodic acid and methenamine–silver–borate solution (Grocott’s stain procedure) (Figure 1) was used to visualize Actinomyces.

Recovery was considered achieved when there was disappearance of symptoms, regression of the inflammatory syndrome, or disappearance of ultrasound or scan image findings. Follow-up varied by doctor (laboratory examinations, imaging, interval between consultations). 

The database was managed using Excel (14.7.7 version, Microsoft Corporation, Redmond, WA, USA) and statistical analyses were performed using R software (3.3.1 version, available online). Statistical analysis was based on Fisher’s exact test for our categorical variables. Values of *p* < 0.05 were considered to denote significant differences.

## 3. Results

### 3.1. Clinical Symptomatology

A total of 27 patients presenting PA were included and analyzed. Patients’ mean age was 49 years (19–86 years) at the time of diagnosis. Among them, 7.4% (*n* = 2) had a state of immunodepression because of advanced breast cancer under chemotherapy (all of them had copper IUDs); 33.3% (*n* = 9) had undergone digestive surgery, including one complicated by pelvic peritonitis, without history of IUD use; and 25.9% (*n* = 7) had undergone gynecological surgery, including one during the month preceding diagnosis (hysteroscopic polyp resection with insertion of a copper IUD). Among the patients, 76.9% (*n* = 20) used an IUD for contraception for a median duration of 9 years (0.1–30).

At the time of the first visit, the patients were all symptomatic, with complaints of pelvic pain (70.4%, *n* = 19), leukorrhea (22.2%, *n* = 6), and/or metrorrhagia (37%, *n* = 10). Only 29.6% (*n* = 8) of the patients were febrile. Other complications, such as digestive and urinary functional signs, recto-vaginal fistula (one case), and a cutaneous fistula (one case) were described. At admission, 72.2% (*n* = 13) presented a biological inflammatory syndrome. In 22.2% (*n* = 2), the CA125 was high. 

### 3.2. Imaging Exploration

A pelvic ultrasound (74%, *n* = 20), an abdominal / pelvic CT scan (67%, *n* = 18), and/or an abdominal/pelvic MRI (22%, *n* = 6) were performed depending on the clinical picture, severity, and availability of resources. Most imaging found tubo-ovarian abscesses and/or infiltrating masses, initially tubo-ovarian of mixed components, +/- necrotic or abscessed. Some pointed to an invasion of the digestive (anterior rectal or right colic flexure) and/or urinary tract with hydroureteronephroses, sometimes bilateral. One case showed a lymphatic invasion with multiple precaval and pelvic lymph nodes. 

### 3.3. Diagnostic Tools

In our study, two thirds of the patients were mildly symptomatic, with no clinical or paraclinical severity criteria, which allowed the first test results (bacteriological or pathological) to be waited upon before starting treatment. Thus, it was possible to propose the diagnosis before any treatment in 66.8% of cases versus 33.3% a posteriori, whatever the diagnostic tools used, thus allowing for adaptation of treatment (Table 1). Several tests were performed per patient, explaining why the number of bacteriological and histopathological tests described herein is greater than the total number of patients.

#### 3.3.1. Pre-Treatment

With bacteriological tools used pre-treatment, only 16.7% (*n* = 1) of the IUDs removed and 22.2% (*n* = 2) of vaginal swabs revealed Actinomyces. In total, 13.6% (*n* = 3) of the bacteriological samples of all types gave positive results. The hemocultures (*n* = 2) and endocervical swabs (*n* = 5) were all negative for Actinomyces. Before the start of any treatment, samples were taken for histopathological analysis: 83.3% (*n* = 5) of the Pap smears were positive. The nine endometrial biopsies were all positive (100%), as was the biopsy of the lesion performed under ultrasound guidance. In total, 93.8% (*n* = 15) of the histopathological samples made it possible to establish a pre-treatment diagnosis of PA (Table 1).

#### 3.3.2. Post-Treatment

For 33.3% patients (*n* = 9), a diagnosis of PID could not be suggested due to the negative results of all the samples (a negative Pap smear for one patient and negative bacteriological samples for the others). 

The diagnosis was made a posteriori, following surgical treatment, thanks either to definitive surgical histopathological specimens in 92.3% (*n* = 12), or thanks to the per-operative bacteriological samples (33.3%, *n* = 4). Whatever the type of sample and the time of its collection, bacteriological analysis only diagnosed actinomycosis in 20.6% (*n* = 7) of cases versus 93.1% (*n* = 27) for histopathological analysis. 

### 3.4. Treatments 

The choice of treatment was made according to the clinical and paraclinical picture, taking into account the degree of infiltration, the possible damage to adjacent organs (digestive tract, urinary tract, lymphatic system), and the area. In the series, one patient had surgical treatment directly without antibiotic therapy. The diagnosis of PA was confirmed by histopathological examination of the surgical specimen. Among the patients, 40.7% (*n* = 11) received only antibiotic therapy. For all patients in this group, the diagnosis was made before any treatment, either by histopathological examination (*n* = 10, 100% positivity), or by bacteriological analysis after a vaginal swab (*n* = 1, 14.3% positivity). A mixed treatment of antibiotic therapy and surgery was carried out in 55.6% (*n* = 15) of cases. Antibiotic treatment was provided for 67 days on average (from 7 days for an endometritis, to 180 days for the most serious cases, with involvement of the adjacent organs of the hydroureteronephrosis type).

Depending on the involvement and choice of treatment, with or without surgery, the duration of antibiotic treatment varied, with a tendency towards reduction in case of surgery. The average duration of antibiotic treatment was calculated for each type of infiltration (Table 2). More than 60% of patients received penicillin A (amoxicillin +/- addition of clavulanic acid). For all users of an IUD, the device was removed.

Surgical treatment differed according to the clinical presentation, from simple drainage-washing of tubo-ovarian abscesses, to an adnexectomy/hysterectomy for infiltrating masses without involvement of adjacent organs, to extensive surgeries of the carcinological type with need for digestive or urologic surgery for infiltrating masses with involvement of adjacent organs. It is noteworthy that a cesarean was required for the case of chorioamnionitis, resulting in the premature birth of the child at 26 weeks of amenorrhea. Several patients with infiltrating masses with involvement of adjacent organs needed repeated surgeries to reach complete recovery.

### 3.5. Follow-Up

Out of all the patients, two of them (7%) presented sequelae, despite a combined treatment. One case involved chronic hemodialysis due to chronic obstructive renal failure associated with a stenosis of the rectosigmoid junction following anastomosis resection. The other was a case of recto-vaginal fistula. The mean time to recovery was 63 days (6–249 days). No case of relapse (clinical and/or paraclinical) was observed across all initial treatments combined (antibiotic therapy or not, duration of the antibiotic therapy, surgery or not). Median follow-up was available for only 13 patients; it was 96 days (4–4339).

## 4. Discussion

Our study suggested a very good performance of endometrial biopsy to diagnose PA, which could avoid surgical diagnosis. Indeed, it was often difficult to establish the diagnosis of PA because patients’ clinical presentation was heterogeneous and non-specific. The invasive potential of the infection into adjacent organs and the possible increase of CA125 tumoral markers [18] cause frequent confusion with advanced-stage ovarian neoplasia [15,16,19]. In our study, some of our patients had first been referred to and cared for in a cancer center before the diagnosis was determined. The prolonged use of an IUD, especially a copper device, could support diagnosis [9]. No duration threshold had been established; a mean of 7 years [10] or 8 years was suggested in the meta-analysis of Fiorino [20], who looked into the association of IUD use and development of the disease. This risk was explained by the production of a biofilm by the Actinomyces that prevents the toxicity effect of the copper and reduces the efficacy of antibiotics [21]. In our study, for those patients who had never used an IUD, we found a context of immunodepression and gynecological or digestive surgery, as described previously [11,22].

Due to the few studies that have been performed, it was difficult to know which diagnostic test to use. Our study showed that the histopathological examination allowed a pre-treatment diagnosis of PA in 93.8% of cases. Although minimally invasive X-ray- or ultrasound-guided biopsies were good examinations, as reported by Pombo [23] and Lee [24], the Pap smear and endometrial biopsy were the most routinely accessible tools and could be performed at the first consultation. In our study, endometrial biopsy with the Pipelle de Cornier made diagnosis possible in 100% of the cases when it was performed using differential staining with histological analysis [18,25]. We noted that the endometrial biopsy was invasive but acceptable with the Pipelle de Cornier (no real pain, only a few cramps or spasms described). Although its specificity and sensitivity could not be evaluated in our study, one could infer that the endometrial biopsy was more specific than the Pap smear because of the sterility of the uterine cavity. In fact, asymptomatic vaginal and cervical carriage of Actinomyces on an IUD is frequent, with a prevalence from 1.6% to 44% [7]—13.7% according to Kalaichelvan [4,20]. Westhoff [26] and Lippes [27], in their retrospective studies, showed that the Pap smear had a poor sensitivity and specificity, as well as a poor positive predictive value. The Actinomyces found on the Pap smear were in no case predictive of the development of PID, and did not confirm the diagnosis. According to Kim et al. [8], no PID due to Actinomyces occurred following the identification of ALO on a Pap smear. Removal of the IUD and beginning of antibiotic therapy had no impact on the development of the disease and, therefore, is not recommended in the case of fortuitous discovery on a Pap smear [4]. 

Few studies have evaluated pre-treatment diagnostic methods in current practice, particularly endometrial biopsy, but our study seemed to confirm the results of Matsuda [18]. This retrospective study including nine cases of PA showed a 66.7% detection rate by cytology before treatment, with all methods included (IUD, endometrial, and cervical). It was clearly shown in our study that bacteriological analysis had no place in the diagnosis of actinomycosis, with a detection rate of 13.6%, thus supporting the strong rate of negative sample results (5.5% to over 50%) reported by Boyanova [2] and Valour [28] in their reviews of the literature, all locations included. These results are due to the anaerobic nature of the bacteria and the difficulty of their isolation from their non-sterile milieu [2,28,29]. Moreover, some molecular methods allowing the identification of the type of Actinomyces are in the process of evaluation (Flurorescence in situ hybridization (FISH), sequencing, real-time polymerase chain reaction (PCR), 16S rRNA gene sequencing, DNA sequencing [30,31,32], and matrix-assisted laser desorption ionization time-of-flight mass spectrometry (MALDITOF MS)) [33].

Finally, there were no recommendations on the choice of treatment. Most studies suggested a combination of antibiotics and surgery. Our study showed that an early diagnosis allowed treatment exclusively with antibiotics, including for severe actinomycosis, with good efficacy and a low rate of complications (7.4%). There was not any case of relapse. The duration of antibiotic treatment varied according to the clinical evolution. This result was in line with a case described of PA extended to the serosa and to the lombo-aortic lymphatic chain. After a pre-treatment diagnosis, the patient was successfully treated with antibiotics exclusively for 6 months (10 million IU/ day of penicillin G IV for 2 weeks, then amoxicillin PO 500 mg × 3/d), without recourse to surgery [34]. Previous studies have reported the need for prolonged antibiotic treatment at high doses because of the seriousness of the infection and a significant tissue fibrosis (penicillin: 2 to 6 weeks IV to continue for 6 to 12 months by oral route). It has not been possible to conduct any prospective studies to date, given the very low prevalence of the disease. Our study showed a tendency to reduce the duration of antibiotic therapy, variable in relation to the extent of the disease, with a recovery rate of 100%. The study of Chiesa-Vottero described the treatment of seven cases of PA diagnosed by endometrial biopsy. Four patients were treated exclusively with antibiotic therapy for 10 to 30 days, two patients had a combined treatment of surgery/antibiotic therapy for between 30 and 45 days, and one patient was treated exclusively with surgery [17]. Actinomyces species do not produce beta-lactamase, so there is no need to widen antibiotic therapy by adding a beta-lactamase inhibitor. Various studies have shown a considerable level of resistance to metronidazole and macrolides, variable according to the strain identified [35]. The benefit of exclusively antibiotic therapy is the reduction of morbidity due to surgery being performed. It was not unusual in the examined studies to find extensive surgeries with associated involvement of the digestive or urinary systems. The retrospective study by Marret [19] compared 11 cases of PA to 58 other cases described in the literature; it reported 45% of extra-pelvic surgeries. These reports highlight why some authors argue for reserving surgery for cases resistant to antibiotics or otherwise complicated. 

Because of the very low incidence of PA during the collection period (19 years) and the lack of standardized international recommendations, the diagnostic and therapeutic course of action was very heterogeneous in our series. Nevertheless, this is the most significant study to date dealing with diagnostic tools used pre- and post-treatment. The review emphasized the advantages of the endometrial biopsy, an easily accessible tool that is widely used in practice. We suggest that if you suspect a PA, you should write “urgent” on the pathological form. It will then take 24–48 h to get a result and to adjust the treatment accordingly. Use of such biopsy would allow modification of therapeutic practices, potentially reduce the morbidity/mortality associated with the disease and its treatment, and help to avoid diagnostic surgeries. A study of a multicenter cohort would be interesting to better analyze the different statistical characteristics of endometrial biopsy and to evaluate the different strategies used for the treatment of actinomycosis. 

## Figures and Tables

**Table 1:** Evaluation of diagnostic tools used pre- and post-treatment in the general population (*n* = 27), then according to the type of treatment.

	Pre-Treatment Diagnosis: N = 18 Patients			Post Treatment Diagnosis: N = 9 Patients		
	***Successfully Diagnosed from Bacteriological Samples***	***Successfully Diagnosed from Histopathological Samples***	***p-Value***	***Successfully Diagnosed from Bacteriological Samples***	***Successfully Diagnosed from Histopathological Samples***	***p-Value***
**General population** **N = 27 patients**	3/22 (13.6%)	15/16 (93.8%)	**<0.001**	4/12 (33.3%)	12/13 (92.3%)	**<0.001**
**Surgery** **N = 1 patient**	/	/	/	/	1 / 1	/
**Antibiotics** **N = 11 patients**	1/7 (14.3%)	10/10 (100%)	**<0.001**	/	/	/
**Surgery + Antibiotics** **N = 15 patients**	2/15 (13.3%)	5/6 (83.3%)	**<0.001**	4/12 (33.3%)	11/12 (91.7%)	**<0.001**

**Table 2:** Duration of antibiotics, relapses, and complications by type of infiltration within the general population (*n* = 27) and by type of treatment.

	Total Population, N = 27				Surgery, N = 1				Antibiotic, N = 11 (40.7%)				Surgery + Antibiotic, N = 15 (55.6%)			
	N	Duration ATB (days)	Relapses (n,%)	Complications (n,%)	N	Duration ATB (days)	Relapses (n,%)	Complications (n,%)	N	Duration ATB (days)	Relapses (n,%)	Complications (n,%)	N	Duration ATB (days)	Relapses (n,%)	Complications (n,%)
**Type of** **infiltration**																
*Endometritis*	3	9	–	–	–	–	–	–	3	9	–	–	–	–	–	–
*Tubo-ovarian abscess*	8	20	-	1	1	–	–	–	2	18	–	–	5	21	–	1
*Infiltrating mass without adjacent organ involvement*	7	104	–	–	–	–	–	–	3	135	–	–	4	73	–	–
*Infiltrating mass with adjacent organ involvement*	7	128	–	1	–	–	–	–	2	180	–	–	5	77	–	1
*Chorioamnionitis*	1	42	–	–	–	–	–	–	–	–	–	–	1	42	–	–
*Unknown*	1	21	–	–	–	–	–	–	1	21	–	–	–	21	–	–
**Total**	27		–	2 (7.4%)	1	–	–	–	11	–	–	–	15		–	13.3%

ATB: antibiotic treatment.
